# Cell wall dynamics: novel tools and research questions

**DOI:** 10.1093/jxb/erad310

**Published:** 2023-08-04

**Authors:** Luis Alonso Baez, Laura Bacete

**Affiliations:** Institute for Biology, Faculty of Natural Sciences, Norwegian University of Science and Technology, 5 Høgskoleringen, Trondheim, 7491, Norway; Institute for Biology, Faculty of Natural Sciences, Norwegian University of Science and Technology, 5 Høgskoleringen, Trondheim, 7491, Norway; Umeå Plant Science Centre (UPSC), Department of Plant Physiology, Umeå University, 901 87 Umeå, Sweden; McGill University, Canada

**Keywords:** Biophysics, cell wall composition, cell wall structure, live-imaging, mechanics, plant cell wall

## Abstract

Years ago, a classic textbook would define plant cell walls based on passive features. For instance, a sort of plant exoskeleton of invariable polysaccharide composition, and probably painted in green. However, currently, this view has been expanded to consider plant cell walls as active, heterogeneous, and dynamic structures with a high degree of complexity. However, what do we mean when we refer to a cell wall as a dynamic structure? How can we investigate the different implications of this dynamism? While the first question has been the subject of several recent publications, defining the ideal strategies and tools needed to address the second question has proven to be challenging due to the myriad of techniques available. In this review, we will describe the capacities of several methodologies to study cell wall composition, structure, and other aspects developed or optimized in recent years. Keeping in mind cell wall dynamism and plasticity, the advantages of performing long-term non-invasive live-imaging methods will be emphasized. We specifically focus on techniques developed for *Arabidopsis thaliana* primary cell walls, but the techniques could be applied to both secondary cell walls and other plant species. We believe this toolset will help researchers in expanding knowledge of these dynamic/evolving structures.

## Teaser

This review offers a comprehensive guide to the latest techniques for understanding the dynamic, active, and diverse nature of plant cell walls, highlighting non-invasive and live-imaging strategies.

## Introduction

Dynamic processes are those related to forces in action. They allow living organisms to change and adapt over time. The processes include evolution, natural selection, and learning. Thus, they are at the very core of life itself.

Plants are sessile organisms, but this does not limit their capacity to develop highly dynamic structures. One of the structures is the plant cell wall. The cell wall plays a vital role in the function and physiology of the plant cell: it provides structural support, protects the cell from internal and external damage, regulates the exchange of materials between the cell and its environment, and is involved in growth and developmental processes (Anderson and Kieber, 2020; B. Zhang *et al.*, 2021). This is only achieved through a fine regulation of biosynthesis and remodelling, allowing the integration of internal and external cues in the plant developmental plan. The ability of plant cell walls to adapt to changing functional requirements during development and interactions with the environment can be described with the term plasticity. Plasticity is also reflected in the diverse composition of their cell walls. The specific makeup of these cell walls can vary greatly, not just between different species of plants, but also among different tissues within a single plant, at various stages of development, and in response to environmental or stress conditions (Knox, 2008; Popper *et al.*, 2011; Bacete *et al.*, 2018). This diversity is even observed on a microscopic scale within individual cells (Dauphin *et al.*, 2022). Yet, this incredible versatility arises from the dynamic assembly of the same fundamental building blocks to form structures tailored to the specific needs of the plant.

Given the dynamic nature of plant cell walls and cell wall plasticity, studying the processes that govern their adaptations to various situations is crucial. To fully understand these processes, we must consider two main aspects: the underlying mechanisms driving these changes and the spatiotemporal scales at which they occur. A comprehensive approach goes beyond merely observing changes and seeks to uncover how and why they happen. Furthermore, we must focus on a temporal scale that allows the organism to adapt to the situation that triggered the change, while still being observable. To this aim, *in vivo* measurements, which involve investigations performed within a living organism, are especially valuable when they enable live measurements, capturing real-time data on the organism’s response to stimuli. This approach contributes significantly to our understanding of cell wall dynamics, shedding light on how plants adapt their structures and regulate their functions. However, *in vivo* measurements are often limited by the characteristics of plant samples since they require small samples, few cells, or cell cultures, in contrast to the larger and more complex nature of most plants. While microscopic analysis may benefit from these simplified conditions, certain procedures, such as tensile tests, usually excel with larger specimens. Particularly in the case of model plants such as Arabidopsis, their small size can be a restrictive factor. This size constraint influences the choice of suitable methods for studying dynamic processes in cell walls. Beyond these considerations, interpreting results, especially from mechanical tests, can become challenging due to the layered complexity of tissues and the variable geometries of plants.

In this review, we aim to describe the advantages and limitations of several techniques used to study cell wall dynamics *in vivo*. Nevertheless, a few techniques that do not allow for *in vivo* measurements are also mentioned because of the unmatched resolution or information obtained. Thus, we aimed to provide a full repertoire of techniques that can fully interrogate the diverse chemical and mechanical properties of plant cell walls. The current and future development of these methods will open new and exciting perspectives in the study of cell walls during dynamic processes, such as growth, development, and interaction with the environment. We have outlined in Box 1 several particularly interesting research questions and technical challenges that are associated with these methods. This knowledge will not only benefit cell wall experts but will also inform general plant biologists, biophysicists, and other researchers interested in understanding the dynamic and adaptable nature of plant cell walls.

Box 1. **Research questions and technical challenges in cell wall research**

**Outstanding biological questions**

- What is the biological reason behind the diversity of cell wall composition and structure in different tissues and developmental stages?
- How do the different cell wall building blocks interact with each other?
- How anisotropic are cell walls and what are the effects on cell wall mechanical properties?
- How does mechanical stimulation of cell walls signal to growth and development? What are the magnitudes of the relevant forces?
- How can mechanical properties measured with different techniques, which give different results, be reconciled into a unified biological trait?
- How do cell wall composition and structure determine mechanical properties of plant tissues such as cell wall stiffness and viscosity?
- What are the structural determinants of cell wall mechanics?
- How are mechanical forces transmitted along cell wall components?
- How do cell wall composition and mechanical properties translate into agronomically important traits such as yield?
- How do tissue-level measurements relate to individual cell mechanisms?

**Outstanding technical challenges**

- Precise predictions of cell wall composition from unlabelled images.
- Implementation of realistic time-dependent cell wall simulations incorporating mechanical and chemical perturbations.
- Measurements and quantifications in growing tissues.

## An intricate network: composition and interactions of cell wall components

The plant cell wall stands as a sophisticated and complex network which is far from being completely understood. It comprises a diverse range of components, each playing a role that is as structurally essential as it is dynamic. Cellulose, hemicelluloses, pectins, and various proteins converge into a structure that is resilient, yet adaptable. Recent reviews can provide an excellent overview of primary (Cosgrove, 2022) and secondary (Zhong *et al*., 2019) cell wall composition and structure. Hence, here we will only provide a summarized version to refresh the advanced reader’s memory and offer an overview to newcomers to the topic.

Cellulose is the main cell wall component. This polysaccharide, composed of linear chains of β-1,4 linked d-glucose units, forms microfibrils that aggregate into larger macrofibrils. These structures lend the cell wall its incredible strength, rivalling the tensile strength of steel (Gibson, 2012). Guided by cytoskeletal elements, these cellulose microfibrils provide an architectural framework for the cell wall, largely determining its mechanical properties (recently reviewed by Chebli *et al.*, 2021). The cytoskeleton also participates in cell wall assembly by helping to transport cell wall polysaccharides from the Golgi apparatus to the extracellular space. Regulating orientation of the cell wall material and deposition rate can produce cell wall anisotropic properties that are used by plants during their development or defence actions, as described in later sections.

Hemicelluloses, primarily composed of xyloglucan molecules in primary cell walls and of xylan and glucomannan in secondary cell walls, are believed to interact intimately with cellulose microfibrils, although the exact nature of this interaction is still under active investigation (Cosgrove, 2022; Du *et al.*, 2022; Coen and Cosgrove, 2023). Two different models have been proposed, namely the ‘tethered network’ model—which establishes that hemicelluloses such as xyloglucan tether cellulose microfibrils together—and the ‘biomechanical hotspot’ model—according to which cellulose microfibrils form tight contacts with each other with the help of a mixture between xyloglucans and disordered cellulose (Cosgrove, 2022).

Pectins, the third critical player, not only regulate the wall’s porosity and flexibility but also can form ‘egg-box’ configurations in the presence of divalent cations such as calcium. This unique conformation contributes to the wall’s overall rigidity, adding to the importance of pectins in maintaining cell wall architecture. In addition, other cell wall elements, such as expansins, are important for controlling cell wall loosening, probably by affecting the contact between cellulose and hemicellulose—although this still needs to be experimentally validated (Cosgrove, 2016b; Samalova *et al.*, 2022).

**Fig. 1.**
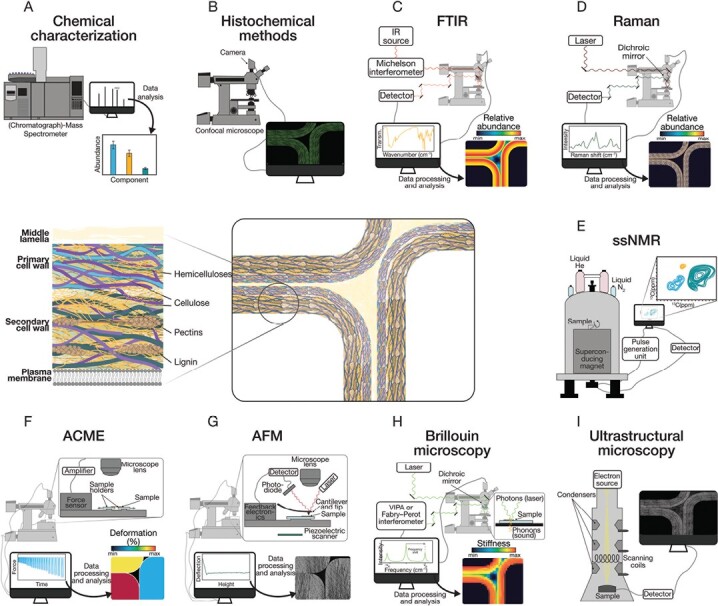
The plant cell wall is a chemically diverse structure composed mainly of polysaccharides (cellulose, hemicelluloses, and pectin), with secondary cell walls also including lignin. These molecules are arranged in a complex 3D structure that gives the cell wall its strength and rigidity. New techniques for studying plant cell walls provide insights into cell wall composition (A–E) and mechanical characteristics (F–I). (A) Chemical characterization provides accurate absolute quantification of the different cell wall components, although with no possibilities of *in situ* studies. (B) Histochemical methods such as using monoclonal antibodies against cell wall epitopes or click chemistry carbohydrate probes allow the location of specific components on the cell walls (e.g. hemicelluloses are marked in green on the figure). (C) Fourier-transform infrared (FTIR) spectroscopy enables relative quantifications of specific structural components (e.g. hemicelluloses) but the resolution is limited. (D) Raman spectroscopy also enables relative quantifications, in this case with higher resolution. (E) Solid-state NMR (ssNMR) allows a high level of detail in the study of chemical composition, but the data interpretation is complicated, and it is not compatible with *in situ* analysis. (F) An automated confocal-microextensometer (ACME) enables the measurement of extensibility and other mechanical properties *in vivo* in individual cells. (G) Atomic force microscopy (AFM) uses a probe (cantilever) to determine cell wall stiffness and topography, and generates an image with nanometric resolution, although it is restricted to the first cell layer. (H) Brillouin microscopy can be used to study mechanical properties in different cell layers with a micrometric resolution. The frequency shift caused by the Brillouin effect is a measurement of stiffness, whereas the amplitude of the signal can be related to viscosity. (I) Ultrastructural microscopy techniques can be used to image macromolecules at submolecular resolution, allowing the observation of cell wall microstructure.

All plant cells have a primary cell wall composed mainly of the above-described components (Fig. 1). The thickness of the primary cell wall in growing cells varies from 50 nm to ~1 μm, depending on the cell type and developmental stage (Derbyshire *et al.*, 2007). Secondary cell walls (Fig. 1) are thicker (≥1 μm; Taiz *et al*., 2022) and function as highly specialized structures that fortify cells that have ceased growing. Predominantly found in tissues responsible for mechanical support, water transport, and defence, these walls are characterized by a higher lignin content, which contributes to their rigidity.

Although we have a good understanding of cell wall composition and structure, resolving the precise interactions of the cell wall components will be an essential prerequisite to understanding global network behaviour and properties.

## The plastic cell wall: tools to study the composition of an ever-changing system

From a biochemical point of view, the cell wall composition is well known for particular plant species, developmental stages, tissues, and even across a single cell wall (Somssich *et al.*, 2016; Gigli-Bisceglia *et al.*, 2020; Molina *et al.*, 2021). However, there is limited holistic knowledge concerning spatio-temporal variations. For instance, a classic biochemical characterization consisting of monosaccharide and linkage analysis (Pettolino *et al.*, 2012) is often performed using whole plants or organs of a particular developmental stage or exposed to different conditions for defined periods of time. Other biochemical characterizations such as oligosaccharide mass profiling (OLIMP), which requires enzymatic digestion followed by massspectrometry on the solubilized oligosaccharides (Günl *et al.*, 2011), have similar drawbacks. On the other hand, histological methods (recently reviewed by Voiniciuc *et al.*, 2018; DeVree *et al.*, 2021) enable a more detailed analysis of cell walls at the tissue and cell levels, but they are limited by sample characteristics. For example, samples that are too thick, such as intact seedlings or plant structures, are unsuitable for most transmission-based microscopy techniques and require slicing and fixing. Other techniques, such as spectrometry-based techniques or advanced microscopy [atomic force microscopy (AFM) or electron scanning microscopy] are restricted to the epidermis and adjacent cell layers. Therefore, techniques that allow *in vivo* compositional analysis are of outstanding interest to detect and quantify the changes in cell wall composition during different growth stages and exposure to different environmental conditions (Fig. 1A–E; Table 1). To illustrate this, we will use three different scenarios from development, response to drought, and plant immunity. We will summarize what kind of dynamic responses occur in each case in primary cell walls, and describe the latest methods used to understand them. Finally, we will discuss which methods we think have strong potential in the future to significantly advance knowledge in the field.

**Table 1.** Experimental techniques to study cell wall dynamics

Technique	Information	*In vivo* analysis (temporal resolution)	Spatial resolution	Advantages	Disadvantages	References
Chemical characterization	Mono-/oligosaccharide composition Glycosidic linkages Decorations	No	None	Quantitative and detailed information	Only provides global information of all cell walls in the sample. Destructive.	Günl *et al* (2011); Pettolino *et al.* (2012)
Fluorescent probes	Relative amount of components Decorations Location	Depending on the technique	180–500 nm	Variety of tools. Defined protocols and relatively easy to use.	Limited information (qualitative). Resolution restricted by the diffraction limit.	Pattathil *et al.* (2010); Knox (2012); Bidhendi *et al.* (2020a)
Click chemistry	Relative amount of components Decorations Location Cell wall network packing density	Depending on the technique [limited to non-Cu(I)-catalysed reactions]	180–500 nm	Better resolution for chemical composition. Non-destructive.	Limited number of non-toxic probes. Resolution restricted by the diffraction limit.	Sawa *et al.* (2006); Anderson *et al.* (2012); Tobimatsu *et al.* (2014); Ropitaux *et al.* (2022)
FTIR	Spectra of IR absorption (determined by chemical composition and arrangement of components)	No	None	High amount of information (polysaccharides, functional groups, linkages, *in muro* organization). Small amount of sample. Non-destructive. Easy to use.	Water interference Data processing is essential and can be complex.	Alonso-Simón *et al.* (2011)
FTIR microspectroscopic imaging		No	1–5 μm	Same as FTIR plus *in situ* analysis.	Resolution restricted by the diffraction limit.	Mouille *et al.* (2003)
Raman spectroscopy microscopy	Raman spectra (determined by molecular composition and distribution)	No	0.5–1 μm	Non-invasive. Label-free.	Long acquisition time. Sample manipulation. Resolution limited by diffraction limit.	Mateu *et al.* (2020)
CARS/SRS		Yes	130–300 nm	Same as Raman but with a lower acquisition time. Allows quantitative analysis (only SRS).	Sample manipulation. Sample fluorescence. Resolution restricted by the diffraction limit.	Zhao *et al.* (2019); Xu *et al.* (2021)
ssNMR	NMR spectra (structure of molecules, chemical composition, and dynamics)	Yes	Atomic resolution	Very high amount of information. Nanometric scale.	Complex information. Requires specific and expensive equipment. Requires NMR-active isotopes or cryogenic temperatures.	Zhao *et al.* (2020, 2021)
Tensile tests	Material deformation (stretching) upon force loading	Limited	Whole tissue	Measurements in living tissues. Measurement of in plane stiffness.	Heterogeneous/non-homogeneous behaviour of cell walls.	Bidhendi and Geitmann (2018a); Suslov and Vissenberg (2018)
ACME		Yes	Single cell	Same as in tensile tests but allowing simultaneous imaging.	Tissues need to be accessible.	Robinson *et al.* (2017)
AFM	Young’s modulus based on the interaction of a thin cantilever with the sample	Possible	1–10 nm	Direct measurements of cell wall mechanics. Label-free.	Limited to first cell layer (epidermis). Invasive.	Krieg *et al.* (2018); Zhao *et al.* (2019)
Brillouin microscopy	Longitudinal elastic modulus based on Brillouin effect	Yes	1–3 μm	3D measurements. Non-invasive. Label-free Short acquisition times.	Indirect measurements of cell wall mechanics.	Caponi *et al.* (2020); Yang *et al.* (2022, Preprint)
FE-SEM	Ultrastructure of surfaces	No	1–5 nm	High resolution.	Limited to surfaces.	Zheng *et al.* (2018, 2020)
Cryo-ET	Ultrastructure of surfaces in cryogenic samples	No	1–5 nm	High resolution. Native conditions preserved.	Imaging requires vacuum conditions and low temperatures. Limited to surfaces.	Sarkar *et al.* (2018); Nicolas *et al.* (2022)
CLEM	Ultrastructure and component’s location	No	3–5 nm	High resolution. Combined fluorescent and electron microscope imaging.	Limited to surfaces. Fluorescence and electron imaging gap.	Chambaud *et al.* (2022)
sNano-FTIR	Spectra of IR absorption (determined by chemical composition and arrangement of components) and topography	No	20 nm	High resolution of chemical composition. Non-destructive. Label-free.	Limited to first cell layer.	Huth *et al.* (2012); Charrier *et al.* (2021)

### Development

The primary cell wall has multiple crucial functions in plant development. It gives shape to newly dividing cells, regulates cell growth and cell cycle progression, controls cell specialization, and responds to external stimuli (Bidhendi and Geitmann, 2016; Voxeur and Hofte, 2016; Gigli-Bisceglia and Hamann, 2018; Anderson and Kieber, 2020). Biochemical and enzymatic modifications of cell walls lead to the loosening, strengthening, extending, or softening of the structure, which is essential for plant development (Peaucelle *et al.*, 2011; Park and Cosgrove, 2012; Cosgrove, 2016a; Coen and Cosgrove, 2023). Consequently, the connection between the composition and structure of plant cell walls is especially close for these processes. In addition to the examples presented here, we will further examine structural changes to the cell wall later in this review.

In this context, pectins are especially relevant. Both pectin composition (i.e. the relative abundance of each of the different pectins) and pectin methylesterification and de-methylesterification play important roles in plant cell growth (Peaucelle *et al.*, 2011; Braybrook and Peaucelle, 2013; Altartouri *et al.*, 2019). Different histological techniques such as propidium iodide staining or oligosaccharide-based probes have been used for *in vivo* labelling of pectins (Bidhendi *et al.*, 2020a). These probes have several distinct advantages over immunostaining (another histological method that we discuss in the next subsection), such as smaller size, better tissue penetration, and higher resolution, the possibility to perform time-course experiments, and easier dual labelling protocols. Furthermore, these probes are suitable for studying the dynamics of (de)methylesterification because they are highly sensitive to changes in methylation and acetylation status. This sensitivity is particularly relevant when considering the ‘egg-box’ model, where different degrees of methylesterification influence the structure and functionality of the cell wall. In one prominent example, Mravec *et al.*, (2014) demonstrated that a chitosan oligosaccharide (COS) probe coupled to Alexa Fluor 488 (COS^488^) binds specifically to homogalacturonan (HG)-containing pectic polysaccharides and oligogalacturonate (OGA) derived from HG. The binding of COS^488^ to pectic polysaccharides decreased with increasing degrees of methylation, and to oligosaccharides with increasing degrees of polymerization. Using the COS^488^ probe, Mravec *et al.* (2014) tracked HG turnover in Arabidopsis root cap cells and found that inhibiting demethylesterification and lowering HG synthesis reduced COS-binding sites and caused defects in cell adhesion. Removing methyl esters created cleavage sites for HG-degrading enzymes that promoted cell separation. This indicates that HG methylation is fine-tuned at the cellular level and may primarily contribute to cell wall stiffening rather than adhesion in mature root cap cells under mechanical stress. However, a drawback of the COS probe is its dependence on ionic strength for binding, which can be influenced by local environmental factors. In a subsequent study (Mravec *et al.*, 2017), the authors developed oligogalacturonide (OG)-based probes (OG7–OG13) to visualize the sites of egg-box formation *in situ*. The OG7–OG13 probes probably do not extensively affect cell wall homeostasis at low concentrations, and their interactions do not rely exclusively on ionic interactions, making them more reliable for studying the dynamics of HG processing. Still, these probes are strongly limited by the type of interactions occurring between carbohydrates.

Another alternative to study pectin turnover is click chemistry labelling. This uses bio-orthogonal chemical reporters, which are analogues to monosaccharides (azido- or alkyne-monosaccharides). They are incorporated into the cell wall target polysaccharide using the cell’s endogenous biosynthetic machinery. Once synthesized and deposited in the cell wall, the polysaccharide containing the reporter can be coupled to an exogenous fluorescent probe through a covalent bond, allowing study of their location and relative amount (Sawa *et al.*, 2006). In Arabidopsis, the first application of this method used a fucose analogue to study pectin organization and dynamics (Anderson *et al.*, 2012). However, a major drawback of this approach is that it uses copper I ions as catalysers, which are toxic for Arabidopsis and damage the wall. More recently efforts have been made to improve the technique by developing copper-free labelling methods (Hoogenboom *et al.*, 2016). To date, there are a limited number of monosaccharide analogues for click chemistry available that have no toxic effects on the cell wall. Last year, Ropitaux *et al.* (2022) described a method using the azido-monosaccharides Fuc-N_3_ and Kdo-N_3_ for *in vivo* labelling of Arabidopsis root cell walls. This method enables the labelling of the pectin polysaccharide rhamnogalacturonan-II (RG-II) and is an example of the future direction required for this field. Interestingly, the regulation of cell wall lignification in distinct cell types, which happens at specific stages during the process of cell differentiation, and its subcellular localization, can be also examined through click chemistry (Tobimatsu *et al.*, 2014; Pandey *et al.*, 2015; Lion *et al.*, 2017).

Cellulose also has a critical role in plant growth, development, and morphogenesis. If cellulose microfibrils are synthesized in a preferential orientation on the cell wall, this region will be reinforced, and the anisotropy of the wall composition will facilitate cell expansion in the direction of minimum stress/stiffness (perpendicular to cellulose orientation) (Green, 1964; Wang and Jiao, 2020). Interactions between cellulose microfibrils and other cell wall components such as hemicelluloses (xyloglucan) or pectins fine-tune wall expansibility. While different models describing the interactions have been proposed (Park and Cosgrove, 2012; Altartouri *et al.*, 2019; Coen and Cosgrove, 2023) they still need to be clarified. Lately, several studies have used solid-state nuclear magnetic resonance (ssNMR) to gain insight into the pectin–cellulose interaction. NMR is a physical phenomenon in which nuclei in a magnetic field absorb and re-emit electromagnetic radiation. This energy emitted has a specific resonance frequency, which depends on the strength of the magnetic field and the magnetic properties of the nucleus. NMR spectrometry uses the observed changes in the nuclear spin states of atoms in a molecule to determine the structure of a molecule, its chemical composition, and its dynamics. In particular, ssNMR can be used to determine the chemical structure, 3D structure, and dynamics of solids and semi-solids at an atomic level, which allows analysis of cell wall composition with high resolution. This spectroscopic method provides a plethora of information on the conformational structure and linkage of polysaccharides, cellulose packing, and water content (Zhao *et al.*, 2020). It has been observed that mutations in genes encoding pectin methyltransferases cause an increase in highly branched arabinan, a particular type of pectin. Moreover, in these mutants, there is a closer association between pectins and cellulose. Specifically, the mutants exhibit a tighter packing between cellulose and pectic backbones (HG and RG-I), pointing to a role for these pectins in the stabilization of cellulose–pectin contacts (Kirui *et al.*, 2021). Despite the high resolution of this technique, a strong limitation is that NMR-active isotopes (e.g. ^13^C and ^15^N) must be incorporated into cell wall polymers to obtain the spectral resolution required for resolving numerous carbon and nitrogen sites in plant cell walls. This inconvenience can be overcome by using the recently developed magic-angle spinning dynamic nuclear polarization (MAS-DNP) method, which enhances NMR sensitivity by tens to hundreds fold. This technique has been used already in poplar (Perras *et al.*, 2017), rice (Zhang *et al.*, 2019; Zhao *et al.*, 2021), and cotton (Kirui *et al.*, 2019) to study polymer composition, structural polymorphisms, and intermolecular packing. However, it cannot be used for *in vivo* studies because MAS-DNP is conducted at cryogenic temperature (~100 K) (Zhao *et al.*, 2021). Other necessary considerations when using that approach include the potential dehydration of the samples due to the centrifugation induced by the spinning of the samples, the heating effect of radiofrequency pulses, and the limitations for maintaining proper physiological conditions (Ghassemi *et al.*, 2022). Furthermore, another limitation is that current NMR methods do not allow the observation of the spatial architecture or the specific location of the different components within the cell wall. Different methods which combine microscopy techniques with NMR have been employed for the study of other extracellular matrices such as biofilms or fungal cell walls (Ghassemi *et al.*, 2022). This is an exciting perspective to further understand interactions between different cell wall components. However, its application to plant cell walls is still to be explored.

### Response to drought

Plant cell walls are essential to how plants respond to changes in their environment. Cell walls serve as a protective barrier against environmental stressors such as temperature fluctuations, UV radiation, and mechanical stress. Moreover, cell walls have a very relevant role in another process that determines drought resistance: stomatal opening. Stomata are small openings or pores found on the epidermis layer of plants. Composed of two guard cells, they are responsible for allowing gas exchange between the plant and its environment, and regulating the water balance of the plant (Meidner and Mansfield, 1968). Fluctuations in turgor pressure within guard cells control the stomatal opening and closure. These fluctuations are, in turn, regulated by the biochemical and mechanical properties of the cell walls, which determine the extent of the morphological changes (Amsbury *et al.*, 2016).

Different histological techniques have been used to identify the components of guard cell walls, which are different from the neighbouring epidermal cells. For example, in Arabidopsis, the guard cell wall is distinguished by a low level of methylated pectins, as detected by the differences in immunofluorescence using the monoclonal antibody LM20—binding to highly methylesterified HG epitopes (Amsbury *et al.*, 2016). An immunostaining using the monoclonal antibodies LM6M—binding short-chain linear arabinan epitopes—and LM13—binding long-chain arabinan epitopes—revealed the relevance of short-chain linear arabinans associated with the presence of RG-I to maintain the flexibility and function of guard cell walls (Carroll *et al.*, 2022). However, although this work was complemented with computational modelling and AFM, it did not fully evaluate the dynamics of the system or the changes in other cell wall components.

Fourier-transform infrared (FTIR) spectroscopy is a non-destructive technique used to obtain the absorption spectra of the molecules in the sample in a range of infrared wavelengths, thus generating a characteristic fingerprint determined by the composition of the sample and by the distribution of the components. When applied to plant cell walls, it can provide information about cell wall composition and *in muro* organization, including the orientation of cell wall polymers (Gierlinger *et al.*, 2008; Kafle *et al.*, 2017). Because of the large amount of data obtained and the complexity of the spectra, the data analysis step is critical (Alonso-Simón *et al.*, 2011). Multivariate analyses are good approaches to extract the maximum amount of information from FTIR data (Gorzsás *et al.*, 2011). An implementation of this technique, FTIR microspectroscopic imaging, permits a detailed characterization of the biochemical composition of specific cell walls *in situ*, although not *in vivo* since this approach requires dehydrated samples. It has been used, for example, to classify cell wall mutants (Mouille *et al.*, 2003), and could be a good fit to study in depth the composition and orientation of cell wall polymers of guard cells. Using microfluidic device such as that used by Devaux *et al.* (2018) to follow enzyme degradation of maize cell walls could be an elegant solution.

### Plant immunity

Plant cell walls are the first barrier that pathogens encounter when they infect a plant. It is for this reason that many pathogens have a plethora of enzymes that attack the cell wall and degrade it. Plants, nevertheless, learned how to recognize this damage and interpret these as a danger signal that triggers plant immunity (Bacete *et al.*, 2018).

Intriguingly, some plants with altered cell wall composition have different resistance patterns against different pathogens. For example, in a recent study, >85% of the analysed Arabidopsis cell wall mutants were either more resistant or more susceptible than wild-type plants to at least one of the pathogens studied (Molina *et al.*, 2021). In this work, all the mutants were examined using monosaccharide and linkage analysis and FTIR to confirm the presence of cell wall alterations, although the specific location or temporal dynamics were not addressed. There are two alternative and not incompatible interpretations for the effect of these alterations in disease resistance. First, the different composition has an effect on the cell wall’s structure, thus complicating or facilitating the access of the pathogen (passive role). Indeed, cell wall reinforcement, for example through the deposition of lignin, is a relevant plant defence response (Bacete *et al.*, 2018). Second, the different composition is reflected in a differential production of damage-associated molecular patterns (DAMPs), which trigger plant immunity (active role). Further biochemical characterization of the differences followed by chromatographic purification has been used to isolate DAMPs present in some of these mutants, which regulate different responses to pathogen attack (Bacete *et al.*, 2020; Mélida *et al.*, 2020). Moreover, DAMPs may prime plants for future pathogen encounters. For example, OGs induce the accumulation of camalexin, which has antimicrobial activity (Gravino *et al.*, 2015). To understand the dynamics of cell wall changes in plant immunity, non-destructive *in vivo* techniques that do not interfere with immunity-related signalling processes are necessary. An example in which these considerations are especially relevant is the use of oligosaccharide probes described above. These molecules could have immunomodulatory activity and affect cell wall homeostasis (Cabrera *et al.*, 2010), which makes them unsuitable for these studies. However, the authors note that only a small amount is required for labelling and should not interfere with the process (Mravec *et al.*, 2017).

Raman spectroscopy, an analysis technique based on the Raman effect or inelastic scattering, uses high-intensity laser light sources that interact with the chemical bonds in the sample. If the energy of the light matches the energy of the molecular vibrations in the sample, a small amount of the energy is scattered. The resulting photons of slightly lower or higher wavelengths can be used to identify the molecular composition of a material (Mateu *et al.*, 2020). Raman spectrometry has been applied to plant cell walls since the 1980s (Atalla and Agarwal, 1986), although some limitations in resolution and the requirement for specialized equipment have marginalized its use. However, there is a newly acquired interest in this technique due to the recent advances in optics and detectors, the new methods to enhance the signal intensity, and the possibility to combine it with confocal microscopy enabling analyses at the micrometre level (<0.5 μm), and thus improving the spatio-temporal resolution (Mateu *et al.*, 2020). Recent improvements have targeted signal levels over spontaneous Raman spectroscopy. For example, coherent anti-Stokes Raman scattering (CARS) focuses the excitation energy specifically onto said Raman mode, whereas stimulated Raman scattering (SRS) uses stimulated Raman gain and loss (Li *et al.*, 2020). Both approaches reduce the scanning times, making them compatible with the observation of live processes. Moreover, SRS can provide quantitative information, although the analytes must overcome a certain threshold abundance (Zhao *et al.*, 2019). Coherent Raman scattering (CRS) is a newly developed protocol tested in Arabidopsis which includes both CARS and SRS to improve the signal-to-noise ratio, and thus is able to avoid the use of resins, therefore reducing the sample manipulation. Interestingly, a sample’s autofluorescence does not interfere with CRS (Xu *et al.*, 2021). Therefore, it could be used as an excellent approach to study changes in cell wall composition upon pathogen infection, following generation of DAMPs and cell wall-reinforcing events.

## A dynamic architecture: tools to evaluate changes in cell wall mechanics and organization

Different techniques can be used to study plant cell wall mechanical characteristics and organization. Here, we will discuss several experimental techniques that provide information with micro- and nanoscale resolution *in vivo*, thus allowing the study of dynamic processes that modulate the viscoelastoplastic characteristics of plant cell walls (Fig. 1F–I; Table 1). *In silico* strategies, which have proven to be vital complements to experimental techniques to predict and examine diverse mechanical processes, are discussed in Box 2.

Box 2. ***In silico* approaches to study cell walls**

Cell shapes in plants are very diverse. They depend on cell wall structure and composition and on the osmotic status of the cell. Due to their complex forms, mathematical models are often used to simulate plant cells dynamics. Modelling has become a useful tool to investigate how biophysical properties of cell walls are modulated under different conditions to produce cell and organ shapes.

Modelling tools go hand in hand with experimental techniques. By using models, complex systems can be reduced to their essential components which makes them easier to understand. Subsequently, hypotheses about the behaviour of the system can be formulated and model validation and refinement be performed. Additionally, the function of mechanisms can be isolated from global signals to understand their individual contribution to complex biological processes (Riglet *et al.*, 2021). Models can be created to analyse different scales. Cell wall components such as cellulose can be modelled at the quantum, atomistic, and molecular level. In the following lines we describe selected approaches developed to investigate cell wall dynamics. The description of the methodologies included in this box is evidently a non-comprehensive source. Readers interested in a more detailed exploration of modelling of plant growth might wish to consult these suggested publications (Geitmann and Dyson, 2014; Morris, 2018; Forterre, 2022; Jensen, 2022).

Coarse grained molecular dynamics models have been successfully used to explore the mechanics and structural properties of cell walls after application of a stretching force (Zhang *et al.*, 2021). Instead of having an all-atoms simulation, the main idea of coarse grained models is to have a reduced representation by grouping functional atoms or molecules into a single particle (Mehandzhiyski and Zozoulenko, 2021). Major drawbacks of these simulations are high computational power, short simulation times (fractions of a second), and the limited number of atoms or molecules that are possible to include in them. An excellent review on cell walls, which describes the general aspects of coarse grained model applications, has been recently published (Cosgrove, 2022).

The finite element method (FEM) is one approach based on continuum mechanics to solve partial differential equations arising from modelling structures and reaction to forces. In biological samples, cell walls/shapes/tissues are discretized and represented as an interconnected mesh of nodes and vertices, and their collective behaviour is modelled (Kennaway and Coen, 2019). FEM can incorporate parameters related to cell walls and mechanical forces to predict changes in cell shape or, based on cell morphology, it can predict biophysical properties. After applying boundary conditions, the effects of perturbations to the system can be calculated for each node. As a result, the displacement of each node together with strain and stress values are obtained. A recent review (Bidhendi and Geitmann, 2018b) extensively described the application of FEM in plants.

Cortical microtubule arrays guide deposition of cell wall materials which in turn determine anisotropic cell wall properties. Therefore, simulations of microtubule alignment and densities are important to understand plant growth. However, the highly dynamic behaviour of microtubule polymerization makes them a difficult subject to study (Elliott and Shaw, 2018). Cytoskeleton imaging has shown that plant cells have a homogeneous array of cortical microtubules across their plasma membrane; however, this aspect was difficult to simulate starting from nucleation sites which generated a positive feedback loop for microtubule nucleation and inhomogeneous arrays in the models. Using a recent model that incorporates a new mechanism in which nucleation was saturated in microtubule-rich regions, acting in opposition to the positive feedback loop, more homogeneous microtubule regions were obtained (Jacobs *et al.*, 2022). Microtubule diffusion rates and the role of their interaction partners in regulating microtubule concentrations are aspects that require future investigations.

Special attention should be given to the time scales and frequencies employed by different techniques to measure dynamic properties. Properties measured in units of seconds or Hertz are practical to describe qualities in the macroscopic scale. However, the spatial scale of cell wall polymer assemblies, both microfibrils and gel-like elements, ranges between 1 μm and 100 μm (Buchanan *et al.*, 2015). Studies on polymers and molecular condensates, which have equivalent dimensions, indicated that measuring dynamic properties in the kilo Hertz to giga Hertz frequencies and in the microseconds to picoseconds time scales are very relevant to understand the intramolecular interactions taking place in these structures (Padding and Briels, 2011; Mitrea *et al.*, 2018; Adichtchev *et al*., 2019; Seifert *et al*., 2021; Keshmiri *et al*., 2023, Preprint). In the following subsections, we introduce techniques that measure dynamic properties in cell walls in these ranges. However, some of them differ in the parameter that is being measured (e.g. AFM and tensile testing compared with Brillouin microscopy, see below) and cannot be directly compared. Therefore, a full description of the method and measured parameters is required when describing sample properties. In addition, we introduce techniques that only allow for static measurements (on non-living samples) but with unrivalled resolution.

### Atomic force microscopy

AFM is a high-resolution microscopy technique that can measure the mechanical properties of the sample with nanometre resolution. The interaction between the tip of a thin cantilever and the surface of the sample generates a mechanical signal that can be used to calculate Young’s modulus, one of the elastic moduli of materials used to relate stress and strain. The definition of the different elastic moduli depends on how forces are applied and how material deformations can occur (Landau *et al.*, 1986). Young’s modulus relates to the axial deformation of a material (strain) when tensile stress is applied along the axis (Allison *et al.*, 2010; Peaucelle *et al.*, 2011; Routier-Kierzkowska *et al.*, 2012; Milani *et al.*, 2013; Caponi *et al*., 2019). The small size and shape (including pyramidal, conical, cylindrical, and spherical) of the cantilever’s probe is the reason for its high resolution compared, for example, with tensile tests. However, its ability to measure mechanical properties at the subcellular level implies that a significant amount of time is required to scan large areas. Additionally, the application of AFM is restricted to directly accessible/surface cell layers. The often invasive nature of the indentation requires that applied forces are controlled to avoid perturbation or damage in living samples, such as potential displacement of cell wall fibres using sharp probes (Braybrook, 2015; Bidhendi and Geitmann, 2019; Asgari *et al*., 2020). In addition, the contribution of turgor pressure to the measurements of elastic constants needs to be taken into account (Beauzamy *et al.*, 2015; Braybrook, 2015; Malgat *et al.*, 2016). Furthermore, the cell samples need to be efficiently immobilized, which limits the application of AFM for live experiments.

AFM has been a relatively widely used method to evaluate mechanical properties of plant cell walls in response to developmental cues, hormonal or enzymatic treatments, turgor pressure, or pectin methylesterification (Weber *et al.*, 2015; T. Zhang *et al.*, 2017; Feng *et al.*, 2018; Song *et al.*, 2020; Wang *et al.*, 2022). However, Young’s moduli values obtained in these experiments are usually lower than those obtained in tensile stretching experiments (see later), or osmotic pressure measurements (where a treatment is applied—typically NaCl, sorbitol or mannitol—to change the turgor pressure inside the cells and cell deformation is quantified to estimate Young’s modulus) (Sapala and Smith, 2020; Seifert *et al.*, 2021). The last two methods allow the measurement of stiffness in the direction of cellular growth, which is presumably more relevant to growth and morphogenesis. In contrast, AFM is usually set to apply an indentation force in the normal direction.

Despite the development of novel AFM modes to study dynamic processes with little perturbation to the samples, their application in time-sensitive mechanical measurements of plant cell walls remain scarce. A recent work by Seifert *et al*. (2021) used multifrequency AFM for the first time on living plants—Arabidopsis hypocotyls—to obtain nanoscale maps of elasticity, viscosity, and time relaxation. The method superimposes multiple frequencies during the cantilever indentation and detects changes in its oscillation parameters, allowing calculation of the dynamic mechanical parameters of the scanned area (García and Herruzo, 2012; Cartagena-Rivera *et al.*, 2015). An adequate model that distinguishes between reversible and irreversible deformations to provide realistic viscoelastic quantification measurements is essential. By setting the cantilever oscillation to frequencies in the kilo Hertz range (time scale of microseconds), Seifert *et al*. (2021) aimed to obtain mechanical measurements directly relevant for plant growth. Young’s modulus values obtained with multifrequency AFM corresponded with reported values using other macroscopic techniques such as tensile stretching and osmotic pressure experiments (hundreds of MPa), in contrast to static AFM measurements which typically produce lower Young’s modulus values (0.1–20 MPa).

### Brillouin microscopy

Brillouin microscopy is based on the Brillouin light scattering effect (i.e. the interaction between incident light and the acoustic waves inside a sample), which leads to a frequency shift of a few giga Hertz in the inelastic scattered light. The sample’s mechanical properties determine the propagation characteristics of the acoustic waves. Hence, the observed frequency shift can be related to the sample’s longitudinal modulus of elasticity, which relates uniaxial stress to strain in the same axis (material is not allowed to expand laterally), allowing quantification of the material’s stiffness (Prevedel *et al.*, 2019). Therefore, Brillouin microscopy presents important advantages in comparison with AFM (Table 1), including the possibility of non-invasive, label-free, and 3D stiffness measurements *in vivo*.

In recent years, this technique has gained relevance for the study of mechanobiology, although it is still relatively new in the plant sciences field. In a pioneering study, Elsayad *et al.* (2016) used Brillouin microscopy to examine the mechanical properties of fluorescently labelled extracellular matrices (a broad concept including plant cell walls) in onion and Arabidopsis. The results confirmed that the method was flexible enough to be applied to different organisms and showed that the hydrostatic pressure in the cell and the viscosity of the cytoplasm impact the mechanical characteristics of plant extracellular matrices. When applied to Arabidopsis, Brillouin microscopy was instrumental to demonstrate that red and far-red signalling modulates the mechanical characteristics of the extracellular matrix (Elsayad *et al.*, 2016). In terms of sensitivity, Brillouin microscopy offered valuable quantitative insights, allowing for precise measurements of these mechanical changes. Further studies have delved into the applicability of this technique, with Altartouri *et al.* (2019) employing it to understand lobe formation in epidermal pavement cells. In an interesting approach, Bacete *et al.* (2022) applied Brillouin microscopy to explore the effects of cellulose biosynthesis inhibition and turgor alterations on root cell walls. This particular study was noteworthy because it demonstrated the technique’s ability to detect changes across the radial axis of the root, including the analysis of inner layers such as the central cylinder, in live samples. In fact, the depth at which Brillouin microscopy can effectively analyse depends only on the sample’s degree of transparency.

Due to the architecture of conventional microscopes, initial Brillouin microscope set-ups needed to rotate the sample to allow stiffness measurements in different directions (Koski *et al.*, 2013; Eltony *et al.*, 2022). Recently, a novel implementation to measure Brillouin scattering at different in-plane angles simultaneously, based on a light dispersive element, was used to measure mechanical properties of periclinal and anticlinal hypocotyl cell walls (Keshmiri *et al*., 2023, Preprint). Measurement of the anisotropic mechanical properties of cell walls with one method highlights the potential of Brillouin microscopy in plant mechanobiology.

While Brillouin microscopy has brought significant advances to plant sciences, it does carry certain limitations (Palombo and Fioretto, 2019; Prevedel *et al.*, 2019; Antonacci *et al.*, 2020; Zhang and Scarcelli, 2021). Firstly, the technique’s ability to probe the high-frequency longitudinal modulus within a sample is not a direct measure of the conventional Young’s modulus. This introduces an additional complexity as there is currently no theoretically established relationship between these two moduli. Nevertheless, empirical correlations have been made to relate the the two moduli with practical success, suggesting that measurements in frequencies different from the biological process time scales are still relevant (Scarcelli *et al.*, 2015). Secondly, the acquisition time of the technique is slower than some other imaging modalities due to the intrinsic weak intensity of the Brillouin signal. Additionally, the instrument’s sensitivity is restricted by the spectral precision of the spectrometer. Despite these limitations, advancements such as confocal Brillouin (Zhang and Scarcelli, 2021) microscopy offer potential solutions, underscoring the promise of Brillouin microscopy for future plant science research.

### Combined spectroscopy approaches: studying composition and structure all at once

Newly developed approaches aim to combine label-free non-destructive techniques that provide information about biophysical characteristics of materials (such as AFM or Brillouin microscopy), with others that provide information about their composition (such as FTIR and Raman spectroscopy/microscopy) (Prats-Mateu and Gierlinger, 2017; Scarponi *et al.*, 2017). These bring a whole new perspective to the study of plant cell walls since they allow simultaneous characterization of both the biochemical composition and mechanical properties in the same sample with high temporal and spatial resolution.

Confocal Raman microscopy and AFM can achieve resolutions to probe composition, structure, and mechanics of single cell walls (<0.5 μm) (Zhao *et al.*, 2019). A correlative application of these techniques was used to track the dynamic changes of cell walls in early wood cells and revealed how the architecture changes under wood compression (Felhofer *et al.*, 2020). Redistribution of cell wall components in the same sample area was then observed by applying compressive forces and looking at the adhesion forces between the AFM tip and the chemical composition of the region probed. Compressed regions contained denser microfibrils and accumulation of lignin compared with open regions which were characterized by porous regions or water-rich regions and fewer denser fibres (Felhofer *et al.*, 2020). These results agreed with Young’s modulus calculations from the slope in graphs of the repulsive force signal following tip snap. A higher Young’s modulus was observed in compressed regions (stiffer) compared with open regions (softer). In addition, this study supports cell wall models highlighting the dynamic interaction occurring at the nanodomains between lignin and other cell wall polymers (Kang *et al.*, 2019).

The diffraction limit is a fundamental limitation of optical systems. This limit is a consequence of diffraction and the wave nature of light, resulting in a diffraction pattern known as an Airy disk (for a uniformly illuminated, circular aperture). According to the Rayleigh criterion, two points can only be resolved when the centre of one diffraction pattern is directly over the first minimum of the other. Consequently, objects smaller than half the wavelength of the incident light cannot be resolved, a phenomenon known as the Abbe limit. Several implementations of the AFM–Raman combination have tried to overcome this limitation, enabling the determination of the biochemical composition of the sample with nanometre resolution. These techniques are based in scanning near-field optical microscopy (SNOM), which places a detection tip in the near-field regime of the sample (within one wavelength) to focus and collect light. Prominent examples are near-field Raman which uses a subwavelength tip aperture to illuminate samples and can achieve resolutions between 50 nm and 150 nm (W. Zhang *et al.*, 2017); and AFM-based tip-enhanced Raman spectroscopy (TERS), which uses an apertureless sharp metal tip positioned at the illumination focus to collect scattered light from the near field to the far field, achieving resolutions up to 1.7 nm (Zhang *et al.*, 2016). However, there are currently no applications of near-field Raman or TERS in plant cell walls, probably because of the low Raman signal, tip manufacturing, and reproducibility issues (Prats-Mateu and Gierlinger, 2017).

In a different setup, a scattering(apertureless)-SNOM combined with an AFM (referred to as nano-FTIR) and equipped with an infrared light source can resolve infrared images, achieving resolutions that only depend on the size of the tip apex (Huth *et al.*, 2012). This instrumentation can be used to obtain mechanical and chemical properties including topography, mechanical phase (sensitive to viscosity), optical amplitude, and phase (related to optical reflectivity and absorption) (Zancajo *et al.*, 2020; Charrier *et al.*, 2021). The chemical composition and the delignification process of primary cell walls in young poplar trees were monitored by measuring local values of dielectric functions of cellulose and lignin at the unprecedented resolution of 20 nm (Charrier *et al.*, 2021). Nano-FTIR has also been used to investigate polysaccharide composition and structure in secondary cell walls and middle lamellae of three varieties of poplar hardwood with different recalcitrance levels (Bhagia *et al.*, 2022). This method has the potential to investigate cell wall modifications induced by physical, chemical, and biological processes.

A particularly interesting emerging approach for studying plant cell wall dynamics arises from the combination of Raman and Brillouin spectroscopic methods. Indeed, both are closely related. Raman spectroscopy uses light to probe the molecules in a material, as described above. The approach is comparable with Brillouin spectroscopy, which uses light to probe vibrations in the sample. In plant cell walls, this combination has been used to examine the mechanical properties and composition of dying *Populus* and *Geranium* leaves (Rakymzhan *et al.*, 2019). However, in this setup, Raman and Brillouin measurements are taken independently. An improved approach that combines both in one microscope, allowing simultaneous measurements, such as the one described by Traverso *et al.* (2015), is certainly an exciting possibility for the future combined studies of plant cell wall composition and mechanical characteristics.

Although the application of these techniques to cell walls can be technically challenging, they are undoubtedly promising strategies for the characterization of their mechanical proprieties and biochemical composition at the nanoscale.

### Advanced microscopy techniques

Traditional light microscopy techniques such as those mentioned earlier do not have the necessary resolution to detect details at the nanometre scale and can only be used to observe cell wall structure or, with the methods mentioned, to study composition. However, they do not provide information to study its organization. In contrast, electron microscopy (EM) techniques, traditionally referred to as ultrastructure microscopy, allow imaging of macromolecules at submolecular resolution (Otegui and Pennington, 2019). As the wavelength of an electron is much smaller than that of a visible photon, EM techniques have better resolution than optical microscopes. In recent years, advances in optical microscopy have led to overcoming the diffraction limit obstacle, at least under some circumstances (Beyond the diffraction limit, 2009). Nevertheless, EM continues to give better resolution compared with light microscopy, although vacuum conditions and sample preparation requirements prevent measurements of live samples.

A conventional scanning electron microscope uses a heated tungsten filament to generate electrons that scan the surface of the sample. An improved version of this setup, field emission SEM (FE-SEM), uses field effect guns. These concentrate low and high energy electrons using a low electrical potential which allows for an improved resolution compared with conventional EM (Zheng *et al.*, 2020). FE-SEM combined with high resolution AFM has been used to explore the fibrillar organization and topography of onion cell walls (T. Zhang *et al.*, 2017; Zheng *et al.*, 2018; Wang *et al.*, 2020).

In electron tomography (or image reconstruction by optical sections), 2D EM projections can be used to reconstruct a 3D structure of an object by imaging at different angles (Ercius *et al.*, 2015; Otegui and Pennington, 2019). This technique can be paired with cryofixation to examine samples in their native state, although potential drawbacks include the possibility of surface artefacts and low accuracy in sample preparation (Ercius *et al*., 2015). Specifically in plant sciences, cryo-electron tomography (cryo-ET) has been used recently to image the nano-architecture of plant cell walls. A study by Sarkar *et al*. (2018, Preprint) used this method to investigate the structure of Arabidopsis stems, revealing detailed measurements of individual microfibrils and their compositions. Their findings indicated that structural imperfections, such as buckling and twisting, in the microfibrils led to a reduction in cell wall stiffness and an increase in ductility, which they suggested could serve as a defence mechanism against rupture after extreme loading. To delve deeper into plant cell structures, Nicolas *et al.* (2022) used cryo-focused ion beam milling prior to cryo-ET, allowing them to access deeper layers in onion scale cells. Their research unveiled a complex interplay between regions with microfibrils and mesh-like regions, presenting interesting trends in mesh occupancy and microfibrillar orientation, with cellulose orientation patterns across successive lamellae following a bimodal and not a helicoidal pattern as previously proposed based on traditional TEM observations (Nicolas *et al.*, 2022). The organization of the microfibrillar orientation is especially noteworthy as it holds significance for plant biomechanics and morphogenesis.

The high-resolution images acquired with EM techniques can be correlated with other techniques which allow for *in vivo* visualization of cellulose microfibril orientation. The fluorescent dyes Calcofluor White, Congo Red, and Pontamine Fast Scarlet 4B target have been used to stain cellulose and observed with polarized fluorescence microscopy (although Calcofluor White also binds to other cell wall polysaccharides and Congo Red also binds proteins) (Prentø, 2009; Anderson *et al.*, 2010). Polarized light is usually not produced by common wide-field microscopes, but most confocal microscopes have plane polarized laser light (Gratton and vande Ven, 2006). These dyes can absorb light and fluoresce depending on light polarization. This phenomenon is called difluorescence or fluorescence dichroism, which has been exploited to observe cellulose microfibrils bound to the dye and oriented parallel to the direction of polarization in root hairs, pollen tubes, and leaf epidermal cells (Thomas *et al.*, 2017; Altartouri *et al.*, 2019; Bidhendi and Geitmann, 2019; Bidhendi *et al.*, 2020a).

Correlative light and electron microscopy (CLEM) is a microscopy technique that combines light (fluorescence) and electron microscope imaging (van den Dries *et al.*, 2022). Although it suffers from the resolution gap between conventional fluorescence microscopy (200 nm) and EM (1 nm), technical advances reduced this difference. CLEM was used to study the formation of plasmodesmata at graft interfaces in Arabidopsis between scion and rootstock hypocotyls of frozen and fixed materials (Chambaud *et al.*, 2022). Using endoplasmic reticulum-retained yellow fluorescent protein (YFP) and red fluorescent protein (RFP) fluorophores, the origin of the observed plasmodesmata was established.

### Tensile testing

The classical test to probe the mechanical properties of materials is tensile testing. The idea is to deform a material by applying a force (stretching), performed by extensometers, which provide information about the mechanical characteristics of materials (Bidhendi and Geitmann, 2019). Uniaxial or biaxial experiments are usually performed by fixing one or two ends of the sample, respectively, and stretching the opposite ends. Two approaches have been commonly used for plants in tensile testing: (i) stretching the material until mechanical failure occurs, allowing the extraction of mechanical properties such as stiffness and strength (Bidhendi and Geitmann, 2018a); and (ii) the creep method, where a constant load is used to stretch the sample and irreversible time-dependent mechanical properties can be detected (Suslov and Vissenberg, 2018). The steps of mounting and clamping or gluing the sample onto the extensometer should be done carefully to avoid building stress before the start of the experiment (Bidhendi *et al.*, 2020b). This limitation applies especially to small samples (as they are more easily damaged) during *in vivo* studies. In comparison with other approaches that measure mechanical characteristics in the normal direction to the surface, tensile testing allows in-plane stiffness and elastic modulus determination (Park and Cosgrove, 2012). These parameters are more relevant for plant cell expansion and directional growth as they reflect the direction of deformation by turgor pressure (Bidhendi *et al.*, 2020b; Majda *et al.*, 2022).

Diverse setups have been used to measure creep and mechanical failure of plant materials at cellular and tissue level to confirm and predict mechanical properties. The automated confocal-microextensometer (ACME) methodology has been used to quantify physical properties in Arabidopsis seedlings with cellular and temporal resolution (Robinson *et al.*, 2017). An interesting outcome of this methodology was the observation of gibberellin treatments increasing cell wall elasticity (Robinson *et al.*, 2017), a key insight for understanding plant growth. Intriguingly, a strain gradient was also discovered along the hypocotyl, hinting at complex, yet to be identified, cell wall properties shaping plant mechanics.

A pitfall in extensometer experiments is assuming that plant tissues are homogeneous (Robinson *et al.*, 2017; Bidhendi *et al.*, 2020b). Plant cells are comprised of cell walls, aqueous cytoplasm, ions, and other macromolecules. In addition, cell wall composition is expected to be heterogeneous, potentially resulting in anisotropic mechanical properties. Intriguingly, extensometer results also show that the longitudinal direction of plant organs is stiffer than the transversal direction (Vanstreels *et al.*, 2005; Bidhendi *et al.*, 2020b). This is contrary to what would be expected for materials expanding, where softer tissues are expected in the direction of principal growth. It was suggested that extensometer experiments quantify tissue-level mechanical properties which differ from cellular properties including cell wall stiffness, cellular geometry, and patterning strength (Majda *et al.*, 2022). In addition, non-linear behaviour has been observed in plant tissues when measuring typical strain–stress curves. It is therefore challenging to calculate Young’s modulus from such curves, as it is typically calculated using the slope of the linear region in strain–stress curves (Bidhendi *et al.*, 2020b). A more sophisticated model would be required to extract mechanical parameters from non-linear strain–stress curves.

## Beyond the wall

During their life, plant cells are exposed to a wide range of mechanical forces. As plant cells are connected to their neighbours via their middle lamella and cell walls, they experience mechanical tension arising in the neighbouring cells during growth and division. In addition, external factors such as wind, dehydration, pathogens, and herbivores create changes in the mechanical environment of cells, which they perceive. How plants sense mechanical forces, transduce them into chemical signals, and produce adequate responses to shape their growth is an area of intense research (Hamant and Haswell, 2017; Bacete and Hamann, 2020; Codjoe *et al.*, 2021; Trinh *et al.*, 2021). In this section, we describe tools to visualize and track how cell walls respond to such forces.

### Molecular probes

Mechanical forces fundamentally influence plant morphogenesis (Trinh *et al.*, 2021). Therefore, to understand how forces are integrated in plant tissues and organs, methods to measure tension changes in cells are necessary. Probes are attractive tools for visualization of specific features as they can be readily applied to the samples before or during experiments. Therefore, probes have great potential to test mechanical forces *in vivo.* A limitation of using probe methods is the need for an independent technique to calibrate the magnitude of forces observed; otherwise, the measurements remain purely qualitative.

Flipper-TR, an emergent tool to measure tension changes in different cellular membranes (plasma membrane, endoplasmic reticulum, lysosomes), has been developed recently (Colom *et al.*, 2018; Assies *et al.*, 2021). It is a probe that adapts the chemistry of push–pull fluorophores, molecules that can be planarized by lateral or axial forces, resulting in measurable changes in the lifetime (Fin *et al.*, 2012; Doval *et al.*, 2014; Dal Molin *et al.*, 2015). Flipper-TR consist of two dithienothiophene flipper molecules, which are twisted with respect to each other due to repulsion forces around the connecting rotatable bond. Flipper-TR signal has been shown to be dependent on both lipid packing and cellular tensions, and was used to detect changes in fluorescent lifetime upon osmotic shocks (Colom *et al.*, 2018). It was also used in a study that investigated the coupling between membrane tension and cell volume during changes in osmotic pressure (Roffay *et al.*, 2021). We are not aware of any reports where Flipper-TR has been used to answer a biological question in plants. If the probe can be efficiently incorporated into the plasma membranes of plant cells, it could be a valuable tool to study the membrane tension in a time-dependent setting.

Microviscosity in different plant cell compartments, including the cell wall and plasma membrane, has been mapped using molecular rotors (Michels *et al.*, 2020). Upon photoexcitation, chemical groups rotate with respect to the rest of the molecule, with the degree of rotation dependent on their interactions with the immediate environment. Rotation causes a detectable change in fluorescence lifetime and intensity of the probe. Molecular rotors were used to detect changes in free volume and lipid packing in the plasma membrane and changes in the network mesh size in the cell wall. Temporal tracking of localized microviscosity during changing environmental conditions is thus possible, as exemplified by experiments where suspension cells were treated with osmoticum at different concentrations. Hyperosmotic shocks (by the addition of mannitol) caused increases in the lifetime of plasma membrane-localized probes, indicating compression forces. In addition, differences in microviscosity were detected *in vivo* using the plasma membrane probe in different root cell types (epidermis root hair-growing and non-growing cells) and using the cell wall-targeted probe in leaf pavement cells in the wild type and mutants with defective cells walls (Michels *et al.*, 2020).

### Microfluidics

**Fig. 2.**
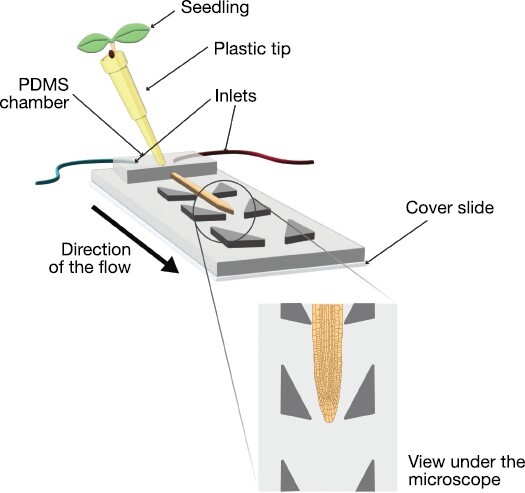
Microfluidics devices allow the study of dynamic responses. The schema represents a basic implementation for a microfluidics device with two inlets, which permits changing of the growing conditions and/or the delivery of specific treatments. The growth of the root tip can be observed under a microscope.

Live-imaging methods allow observation and quantifiation of the evolution of cellular processes over time. Depending on the duration and the conditions of the experiment, live imaging of certain tissues can be performed with very simple setups (Rahni and Birnbaum, 2019). However, to study dynamic responses to environmental stimuli, a tighly controlled system that allows the growing conditions to be changed, to apply specific external cues, and to quantify the induced responses is required. Microfluidics devices (Fig. 2) have been used in plant biology since more than a decade ago due to their capacity to perform live imaging on multiple samples under different conditions (Yanagisawa *et al.*, 2021). Microfluidics devices have been particularly useful to study cell growth dynamics and the behaviour of fluorescent reporters. They have provided insights into how roots respond to sugar levels, hormones, and pathogenic interactions (Grossmann *et al.*, 2011, 2012; Jones *et al.*, 2014; Stanley *et al.*, 2014; von Wangenheim *et al.*, 2017; Fendrych *et al.*, 2018; Liu *et al.*, 2022). However, they have also been employed to study embryo, pollen tube, root hair, leaf, and whole seedling development (Gooh *et al.*, 2015; Vang *et al.*, 2018; Bertrand-Rakusová *et al.*, 2020; Ueda *et al.*, 2020).

Microfluidics devices, offering a fresh perspective on cell wall dynamics, have been instrumental in recent investigations. Root hairs provide an excellent example of a well-established model system to study cellular polarization and coordination of tip cell growth (Ramalho *et al.*, 2022). With microfluidics, Denninger *et al.* (2019) managed to follow root hair tip development over time, discerning a two-phase growth mechanism and the molecular domino effect that triggers root hair formation. Similarly, empirical data obtained from microfluidics, in combination with computational modelling, have been used to answer a long-standing question in biology: the mechanism that signals a cell to halt its growth (Liu *et al.*, 2022). In this work, the authors pinpointed a location in the elongation zone where growth rates begin to slow down, dubbing it the start of growth cessation. To infer cell wall stiffness, cell shrinkage in hyperosmotic solutions was observed using a microfluidics device (drawing on methods by Sapala and Smith, 2020, and devices from Grossmann *et al*., 2011; Denninger *et al*., 2019). The study saw the recorded shrinkage rates as evidence of increasing cell wall stiffness along the root’s longitudinal axis, a perspective diverging from previous models attributing these changes to alterations in cell shape (Sapala *et al*., 2018).

## Conclusions

Traditional techniques to study cell walls have uncovered useful information about cell wall composition and structure, but have fallen short in assessing their dynamic behaviour. The supramolecular network formed among cellulose, hemicellulose, and pectin in primary cell walls has received a lot of attention aiming to understand cell morphology, growth, and responses to environmental signals. In addition, lignin reinforcement in secondary cell walls of certain cell types after differentiation has been the focus of structural and biomass production studies. Nevertheless, it has proven difficult to resolve the organization of cell walls and their chemical and mechanical properties at the nanoscale without modifying the native chemical structure. Quantifying and understanding how fibrils and matrix polysaccharides are produced, interact chemically, and stretch or bend mechanically will enable us to connect the nanoscale behaviour to macroscopic properties, including stiffness, material properties, defence mechanisms, and features optimizing enzyme biomass digestibility.

An intriguing and still largely unexplored question in plant cell wall research is how composition and structure change with developmental stage progression. Changes occurring on the time scale of minutes to days have recently been investigated due to advances in live-imaging technologies, such as microfluidics, and non-invasive techniques, such as Brillouin microscopy and molecular probes, which allow observation and tracking of the evolution of single cell walls within individual organisms. A limitation has been that each technique can test one or few properties of the sample, thus requiring multiple measurements using different methods to fully characterize the walls. Technologies allowing real-time characterization of chemical and mechanical properties within the same measurement would be beneficial to study cell wall dynamics. Another caveat is that most of the current techniques only allow measurements to be made on sample surfaces (epidermis) or on whole tissues. Specific contributions of subepidermal tissues, whose composition is likely to differ from that of the epidermis, to the structural properties of cell walls remain uncharacterized. Enhancing the resolution and performance of non-destructive, contactless techniques will help to uncover the contributions of the different cell wall components to specific plant tissues/cell types. Computational approaches have provided insightful knowledge to predict mechanical properties and cellular morphologies, interpret unlabelled microscopic images, and incorporate results from different sources to guide new experimental steps (Box 2). Simulations could thus play a vital role in integrating knowledge about cell wall mechanics, composition, and structural changes over time, which could help us to understand the mechanisms responsible for cell wall plasticity.

We believe the methods described here form a common toolbox for cell wall studies that could be used to answer challenging questions in the near future including why cell wall composition and structure are so diverse among cell types and how mechanical forces are sensed and transduced in plant cells (Box 1). Furthermore, many of them allow *in vivo* measurements. This opens the door to move from static measurements to study cell walls by following changes occurring in the observed organism induced during growth or in response to environmental cues (during biotic and abiotic stresses).
